# Impact of *Cymbopogon flexuosus* (Poaceae) essential oil and primary components on the eclosion and larval development of *Aedes aegypti*

**DOI:** 10.1038/s41598-021-03819-2

**Published:** 2021-12-21

**Authors:** Ruth Mariela Castillo-Morales, Sugey Ortiz Serrano, Adriana Lisseth Rodríguez Villamizar, Stelia Carolina Mendez-Sanchez, Jonny E. Duque

**Affiliations:** 1 Departamento de Ciencias Básicas, Centro de Investigaciones en Enfermedades Tropicales–CINTROP, Facultad de Salud, Escuela de Medicina, Universidad Industrial de Santander, Guatiguará Technology and Research Park, Km 2 Vía El Refugio, Piedecuesta, Santander Colombia; 2 Grupo de Investigación en Bioquímica y Microbiología (GIBIM), Escuela de Química, Universidad Industrial de Santander, A.A. 678 Bucaramanga, Colombia

**Keywords:** Mechanism of action, Natural products, Entomology

## Abstract

The current study describes the effects of sub-lethal concentrations and constituent compounds (citral and geranyl acetate) of *Cymbopogon flexuosus* essential oil (EO) on the development of *Aedes aegypti*. We treated eggs with 6, 18, or 30 mg L^−1^ and larvae with 3 or 6 mg L^−1^ of EO and its major compounds (citral and geranyl acetate). Citral and geranyl acetate were evaluated at 18, 30, and 42 mg L^−1^ and compared with commercial growth inhibitors (diflubenzuron and methoprene). We measured larval head diameter, siphon length, and larval length. Finally, we examined concentrations of molt hormone (MH) and juvenile hormone III (JH III) using high-performance liquid chromatography coupled to mass spectrometry. All geranyl acetate concentrations decreased egg hatching, while EO altered molting among larval instars and between larvae and pupae, with an increase in the larval length (3 mg L^−1^: 6 ± 0.0 mm; 6 mg L^−1^: 6 ± 0.7 mm) and head width (3 mg L^−1^: 0.8 ± 0 mm; 6 mg L^−1^: 0.8 ± 0.0 mm) compared with the control group. We did not detect chromatographic signals of MH and JH III in larvae treated with *C. flexuosus* EO or their major compounds. The sub-lethal concentrations *C. flexuosus* EO caused a similar effect to diflubenzuron, namely decreased hormone concentrations, an extended larval period, and death.

## Introduction

Each year, more than 2.5 billion people are a risk of being infected with arboviruses like Dengue, Zika, and Chikungunya virus in urban and peri-urban areas transmitted by vector mosquito *Aedes aegypti*^1,2^. Given that there is no effective vaccine for any of these three diseases^3,4^, the principal strategy for mosquito control comprises population decrease using chemical insecticides on juvenile (temephos) and adult (deltamethrin, lambda-cyhalothrin, cyfluthrin, and malathion) stages^4,5^. However, continued application of these products leads to resistant populations as well as unwanted environmental effects^6–10^.

In the search for new mosquito control alternatives, the juvenile stages constitute a suitable target because these stages develop exclusively in aquatic environments and cannot transmit disease to humans. Any interferences or alterations in these environments caused by an external source of hormonal or growth regulation can interrupt the normal life cycle and cause abnormal development in the adult stages^11^. Consistently, a control strategy with notable specificity comprises the use of insect growth regulators (IGR); these differ from conventional insecticides because they cause morphological and physiological changes during development and metamorphosis^4,5^. The most used and studied growth regulators are diflubenzuron (inhibitor of chitin synthesis) and methoprene (juvenile hormone [JH] analogue)^12,13^.

Looking for environmentally friendly products, many research groups have reported that essential oils (EO) have repellent, deterrent, ovicidal, larvicidal, pupicidal, and adulticidal activities against *Ae. aegypti*^14–22^. The authors of these studies have mentioned that EO from the Malvaceae, Asteraceae, Annonaceae, Rutaceae, Piperaceae, and Verbenaceae plant families are metamorphosis inhibitors that cause reproductive alterations in females^23–26^. Researchers have reported that EO from the genus *Cymbopogon* has ovicidal and larvicidal activities against *Ae. aegypti*^27,28^. Moreover, Soonwera and Phasomkusolsil^29^ reported morphological abnormalities juvenile stages of *Ae. aegypti* and *Anopheles dirus* larvae and deformed pupae, as well as incomplete hatching and mortality due to the activity of *Cymbopogon citratus* EO.

**Figure 1:**
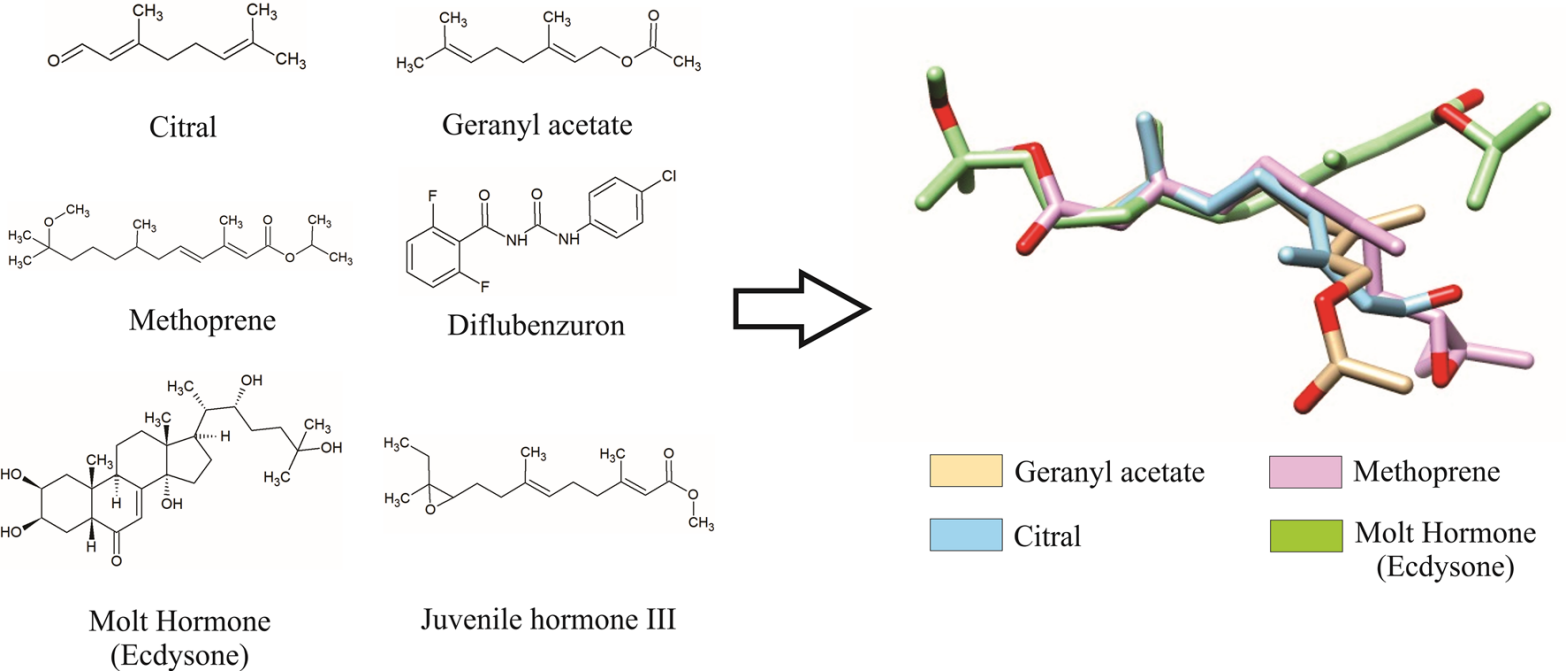
Chemical structures of the major compounds of *Cymbopogon flexuosus* essential oil (citral and geranyl acetate), the positive controls methoprene and diflubenzuron, and moult hormone (ecdysone) and juvenile hormone III. In the coloured molecular model, the chemical structures of each compound have been superimposed (ChemDoodle 6.0 and UCSF Chimera software https://www.cgl.ucsf.edu/chimera/).

Scientific researches have evaluated the activity of *Cymbopogon flexuosus* (Poaceae) EO against *Ae. aegypti* in terms of larvicidal activity, repellence, and dissuasive efficacy using a lethal concentration (LC_50_ = 17.2 mg L^−1^; LC_95_ = 49.9 mg L^−1^)^21,22^. Notably, this EO has compounds structurally similar to commercial molecules with effects like growth and developmental inhibition in insects (Fig. 1), which raises our hypothesis that *C. flexuosus* EO affects the life cycle of *Ae. aegypti*. This work describes the effects of sub-lethal concentrations of *C. flexuosus* EO and its major compounds—citral (a mixture of two isomers, geranial and neral) and geranyl acetate on *Ae. aegypti* eggs and larval development. We also used high-performance liquid chromatography (HPLC) coupled with mass spectrometry to examine the variations in the concentrations of molt hormone (MH) and juvenile hormone III (JH III) in *Ae. aegypti* larvae treated with *C. flexuosus* EO and its major compounds.

## Results

### Ovicidal activity

**Figure 2:**
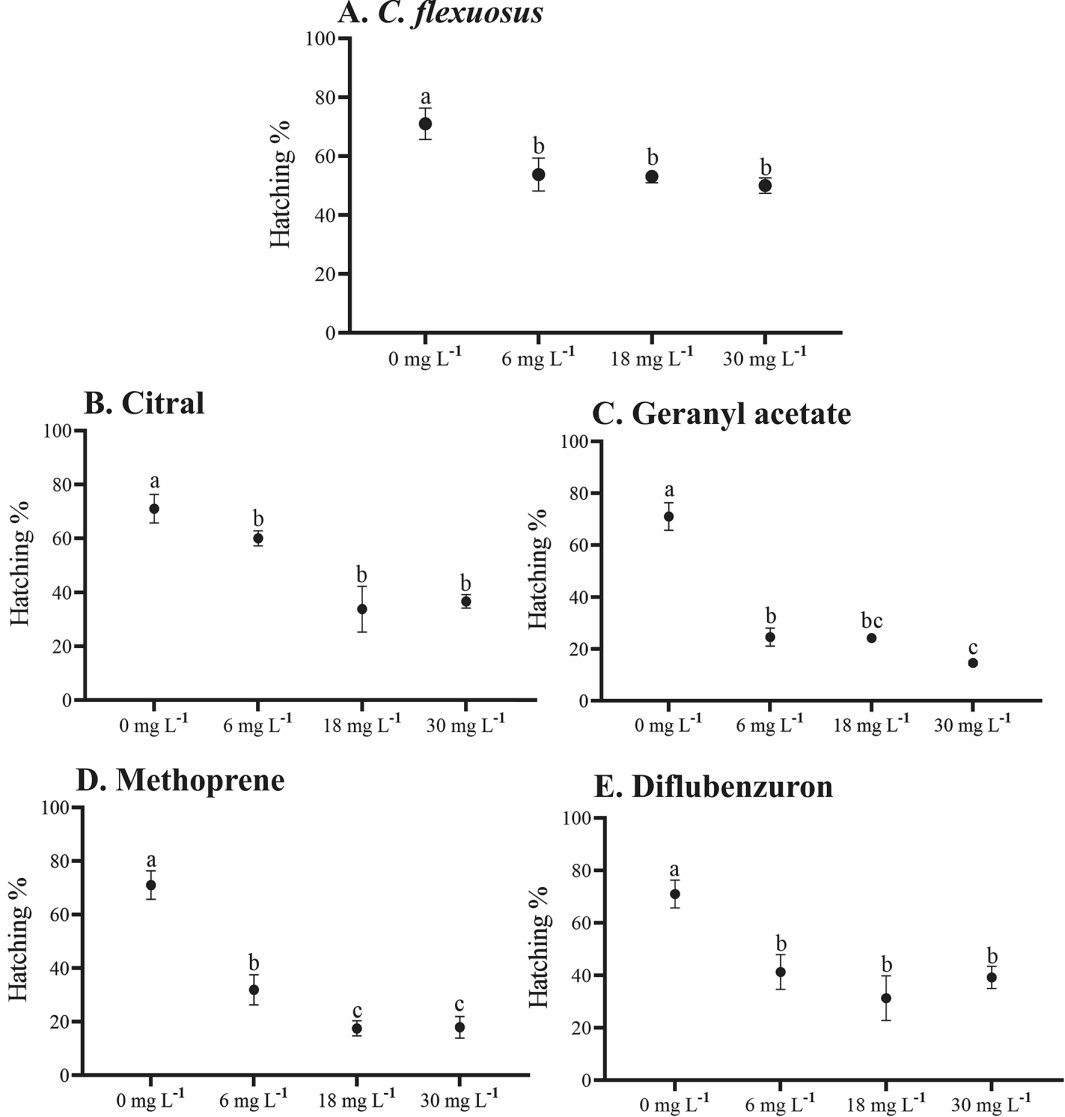
Hatching percentages of *Aedes aegypti* eggs subjected to different concentrations (6, 18, and 30 mg L^−1^) of (**A**) ***Cymbopogon flexuosus essential oil*** (analysis of variance [ANOVA]: F = 7.41776, df = 3, *p* = 0.019205); (**B**) **citral** (ANOVA: F = 17.5349, df = 3, *p* = 0.001232); (C) **geranyl acetate** (ANOVA: F = 77.8167, df = 3, *p* = 0.000034); (**D**) **methoprene** (ANOVA F = 50.9635, df = 3, *p* = 0.000040); (**E**) **diflubenzuron** (ANOVA: F = 15.3481, df = 3, *p* = 0.003203). Different letters (a, b, and c) indicate statistically significant differences between the concentrations of substances and the control group (Tukey’s test, *p* ≤ 0.05).

An ovicidal effect was observed in all treatments, even when compared among concentrations (Fig. 2A–E). Geranyl acetate (75.4%) significantly decreased hatching, and *C. flexuosus* EO (46.2%) exhibited minor ovicidal activity at 6 mg L^−1^.

### Morphologic alterations of the larvae

**Figure 3:**
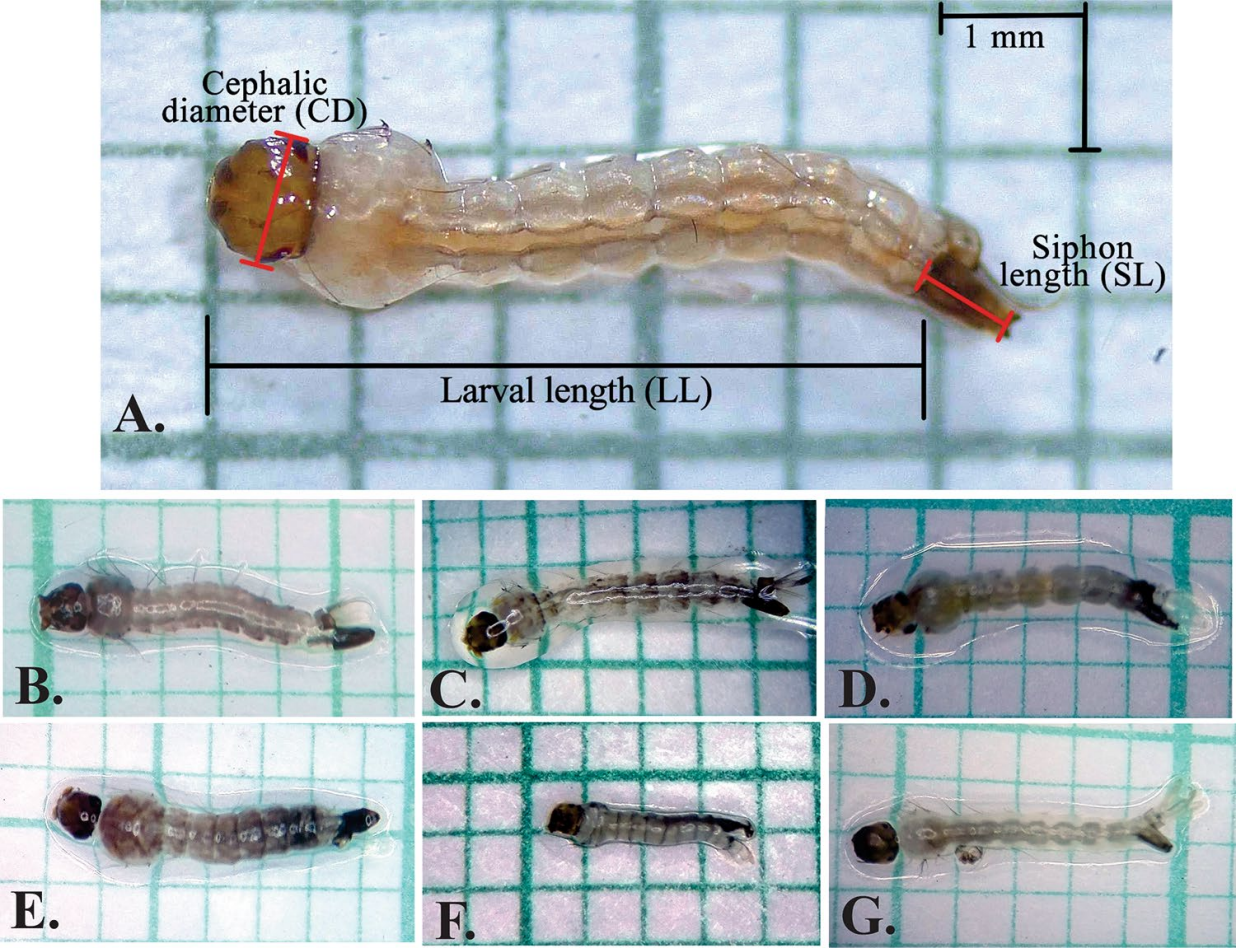
Morphological differences in *Aedes aegypti* larvae subjected to different treatments. (**A**) The morphometric measurements considered are: larval length (LL), cephalic diameter (CD), and siphon length (SL). The photographs include a representative larva from each group: (**B**) control; (**C**) *Cymbopogon flexuosus* essential oil; (**D**) citral; (**E**) geranyl acetate; (**F**) diflubenzuron; (**G**) methoprene.

**Figure 4:**
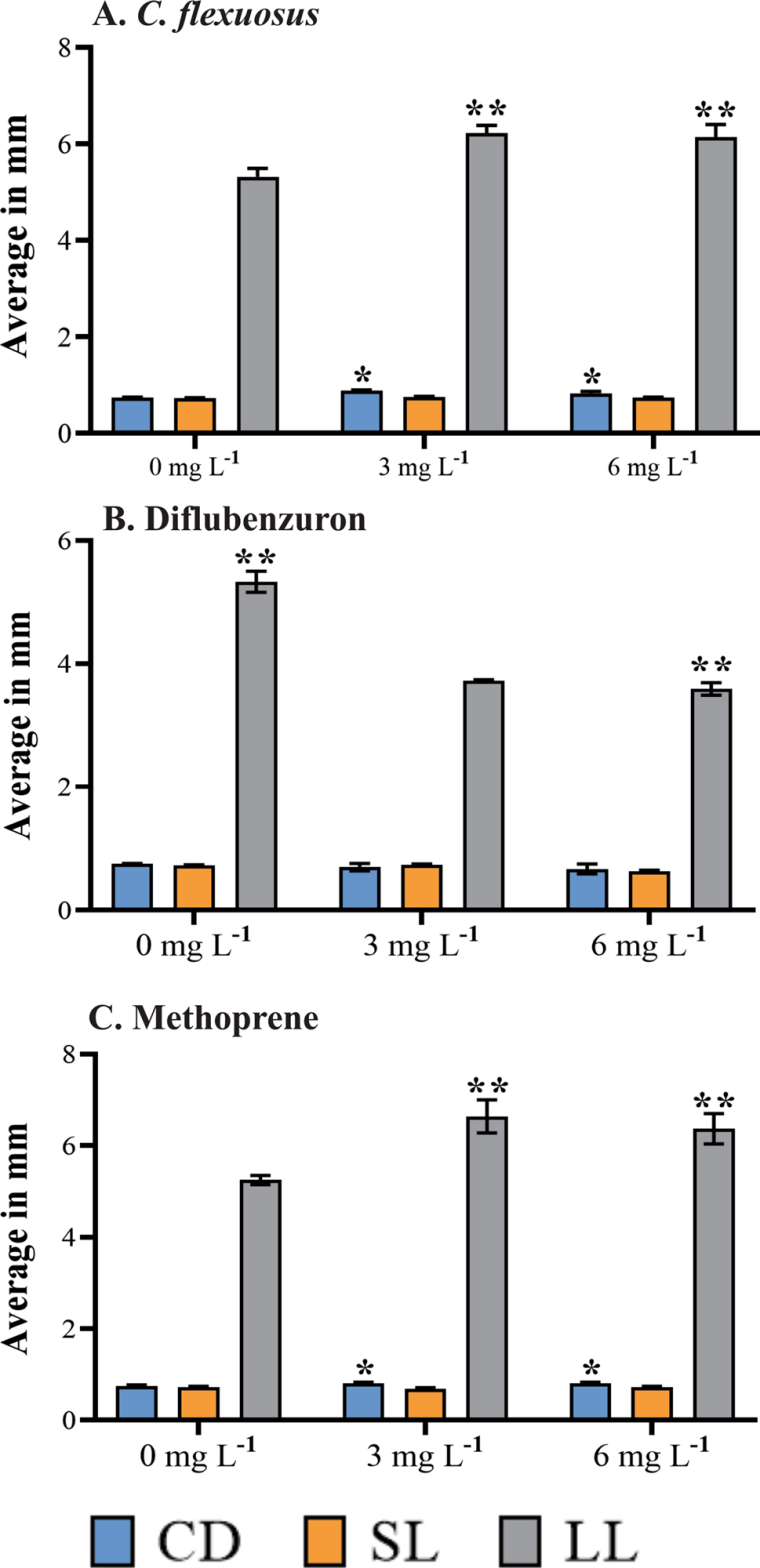
The effect of different concentrations (3 and 6 mg L^−1^) of *Cymbopogon flexuosus* essential oil, diflubenzuron, and methoprene on the development of *Aedes aegypti* larvae. The average larval length (LL), cephalic diameter (CD), and siphon length (SL) are presented in millimetres. (*) Significant differences in CD between the concentrations of each treatment and the control group (Tukey’s test *p* ≤ 0.05). (**) Significant differences in LL between the concentrations of each treatment and the control group (Tukey’s test, *p* ≤ 0.05) by LL. (**A**) ***Cymbopogon flexuosus essential oil*** (analysis of variance [ANOVA]: F = 18.174, df = 2, *p* = 0.002928); (**B**) **diflubenzuron** (Kruskal–Wallis test: H (2, N = 9) = 6.937853, *p* = 0.003171); (**C**) **methoprene** (ANOVA: F = 7.03, df = 2, *p* = 0.002398).

The larvae showed visual changes in larval length (LL), cephalic diameter (CD), and siphon length (SL) in all treatments (Fig. 3A–G). We observed significant increases in the CD and LL when larvae were treated with *C. flexuosus* EO and methoprene (Fig. 4A,C). On the other hand, diflubenzuron only led to changes in the LL. Of note, diflubenzuron decreased the LL whereas the other treatments increased the LL (Fig. 4B).

**Figure 5:**
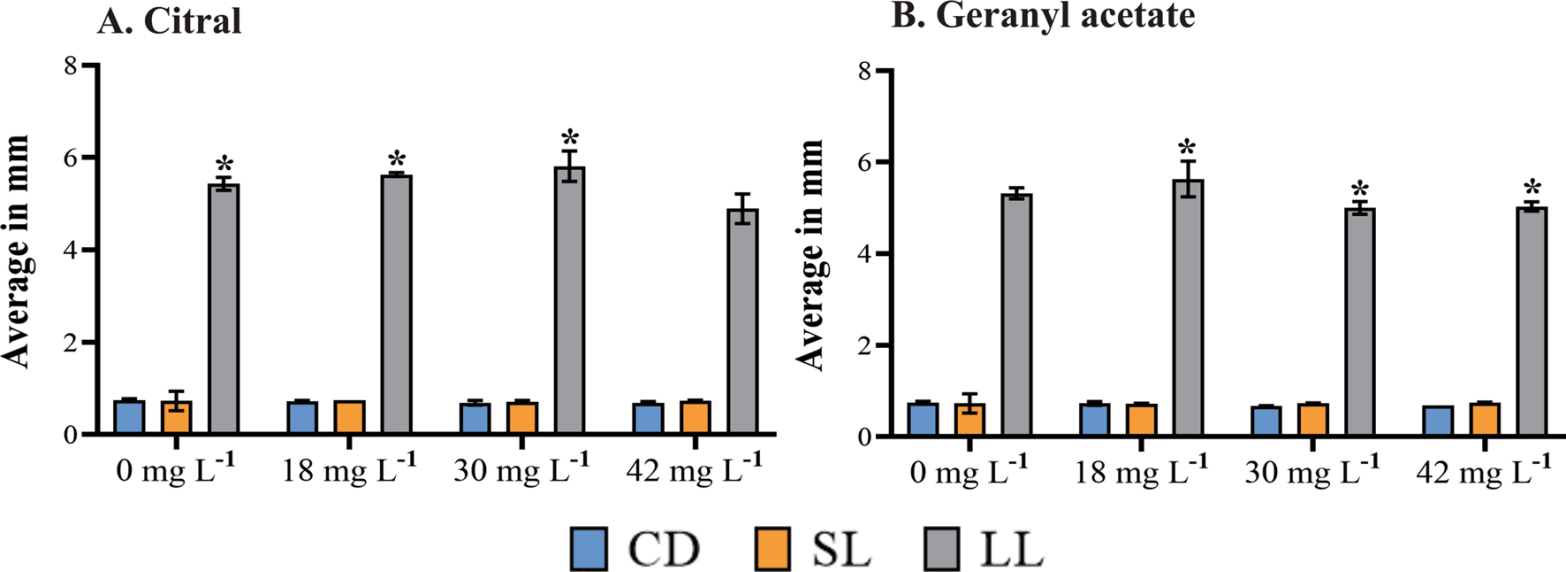
The effect of major compounds of *Cymbopogon flexuosus* essential oil (citral and geranyl acetate) at a concentration of 18, 30, and 42 mg L^−1^ on the development of *Aedes Aegypti* larvae. The average larval length (LL), cephalic diameter (CD), and siphon length (SL) are presented in millimetres. (*) Significant differences in LL between concentrations of each substance and the control group (Tukey’s test, *p* ≤ 0.05). (**A**) **Citral** (analysis of variance [ANOVA]: F = 8.391, df = 2, *p* = 0.007476); (**B**) **geranyl acetate** (ANOVA: F = 8.391, df = 3, *p* = 0.0007476).

Given the average increase in the morphological measures of *Ae. aegypti* larvae treated with *C. flexuosus* EO, we evaluated the effect of its major compounds—citral and geranyl acetate—at concentrations of 18, 30, and 42 mg L^−1^. There were no significant changes in the CD and SL with citral and geranyl acetate compared with the control (Fig. 5). However, geranyl acetate significantly reduced the LL compared with the control (Fig. 5B).

### Inhibition of development

**Figure 6:**
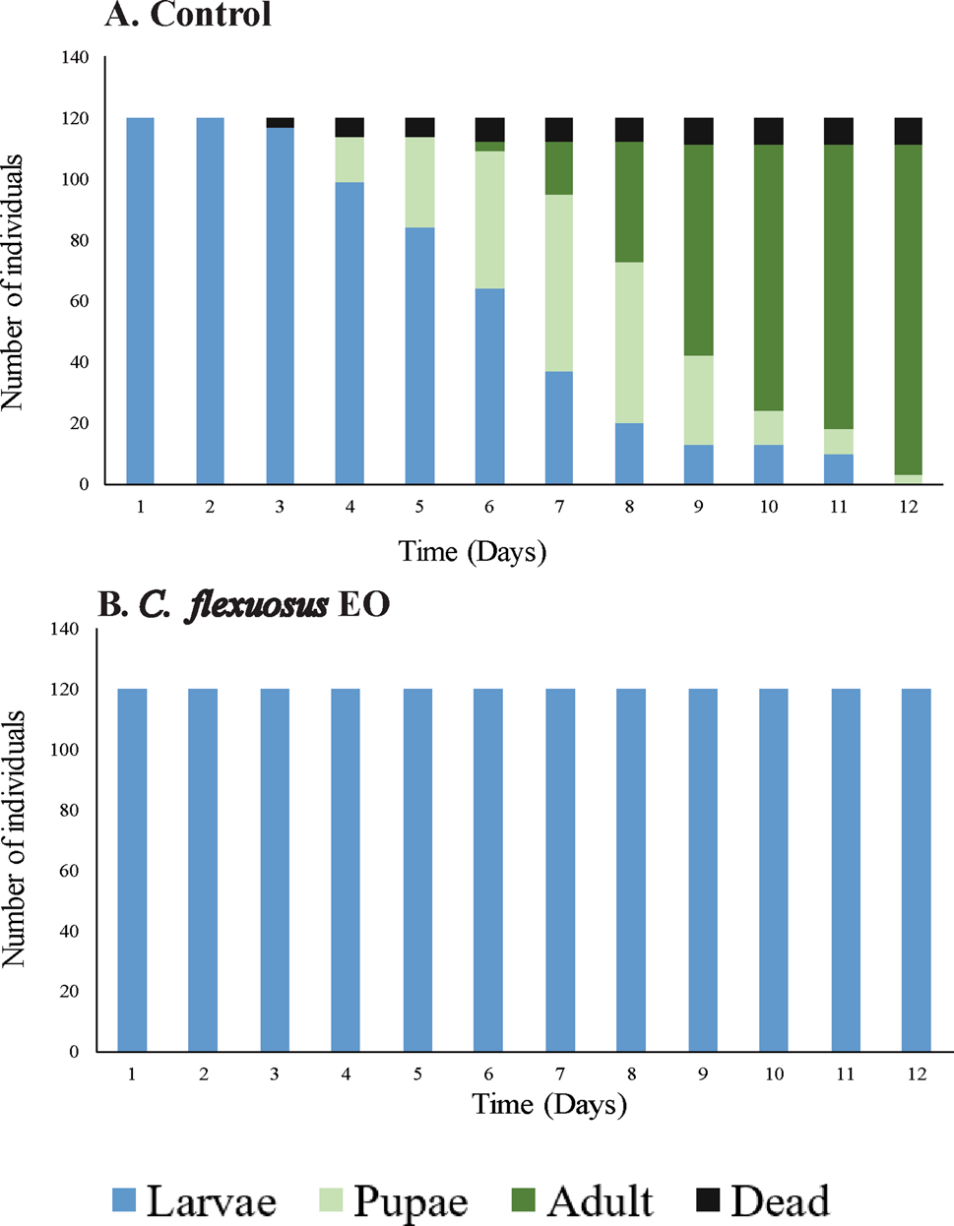
Duration in days of larval development (L3 and L4) as well as pupal and adult stages of *Aedes aegypti*. (**A**) Individuals in the control group. (**B**) Individuals were treated with *Cymbopogon flexuosus* essential oil at a concentration of 3 or 6 mg L^−1^. The results were the same for both concentrations.

**Figure 7:**
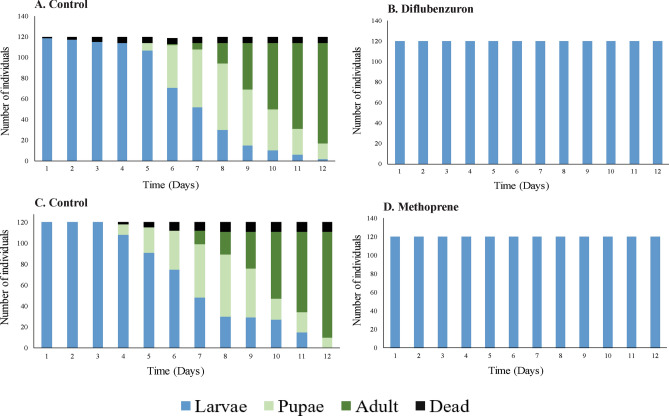
Duration in days of larval development (L3 and L4) as well as pupal and adult stages of *Aedes aegypti*. The individuals were treated with growth regulators at concentrations of 3 or 6 mg L^−1^.

*Cymbopogon flexuosus* EO (3 and 6 mg L^−1^) exhibited an inhibitory effect on *Ae. aegypti* development. None of the L3 larvae reached the pupal stage; they remained in the larval phase for 12 days (Fig. 6B). This outcome was similar to the larvae treated with the positive controls (diflubenzuron and methoprene; Fig. 7B–D). On the other hand, 60% of the larvae in the control group reached the pupal stage at day 7 of development, and the life cycle was completed on day 12 (Figs. 6A and 7A–C).

**Figure 8:**
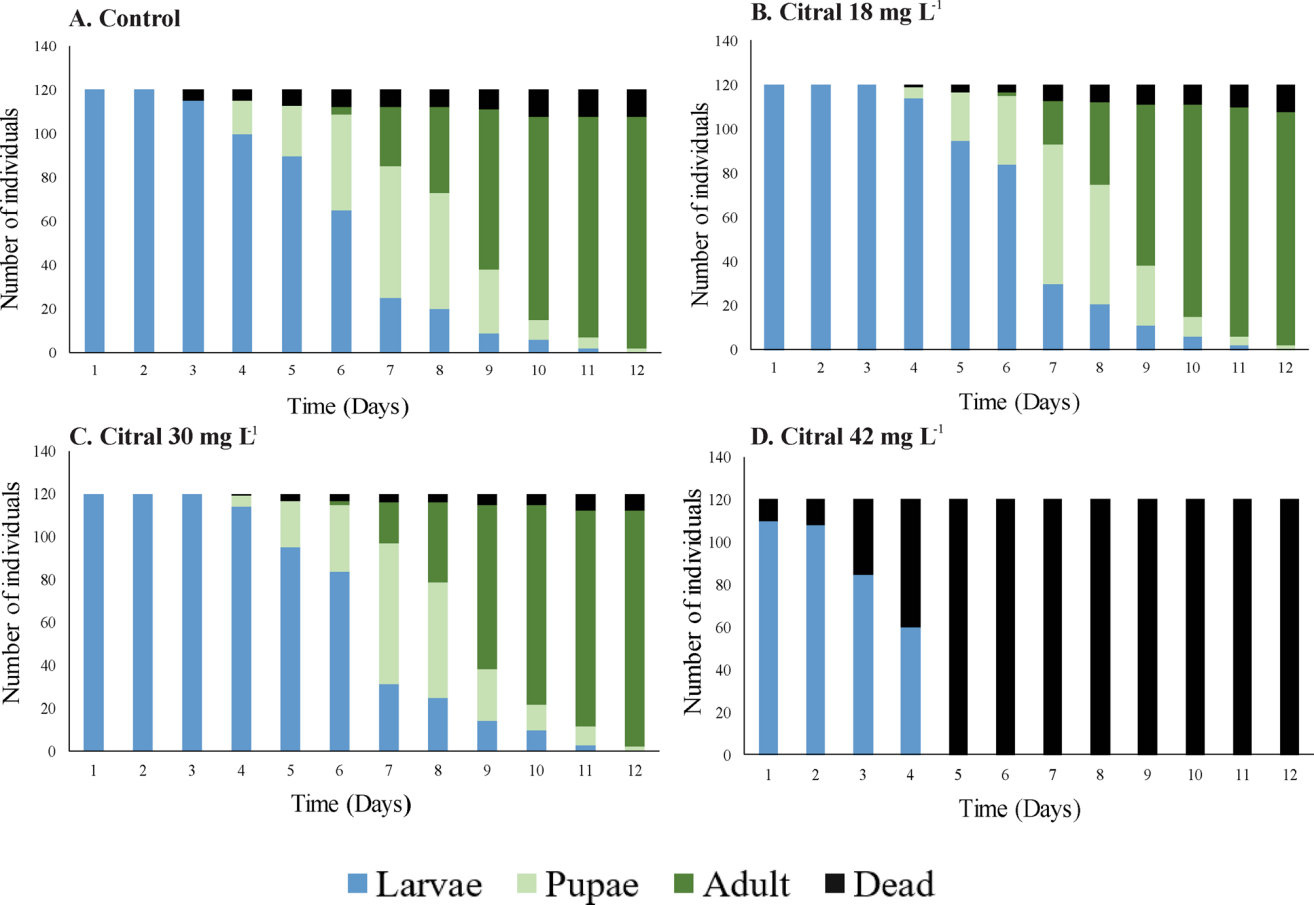
Duration in days of larval development (L3 and L4) as well as pupal and adult stages of *Aedes aegypti.* The individuals were treated with the major compound citral.

**Figure 9:**
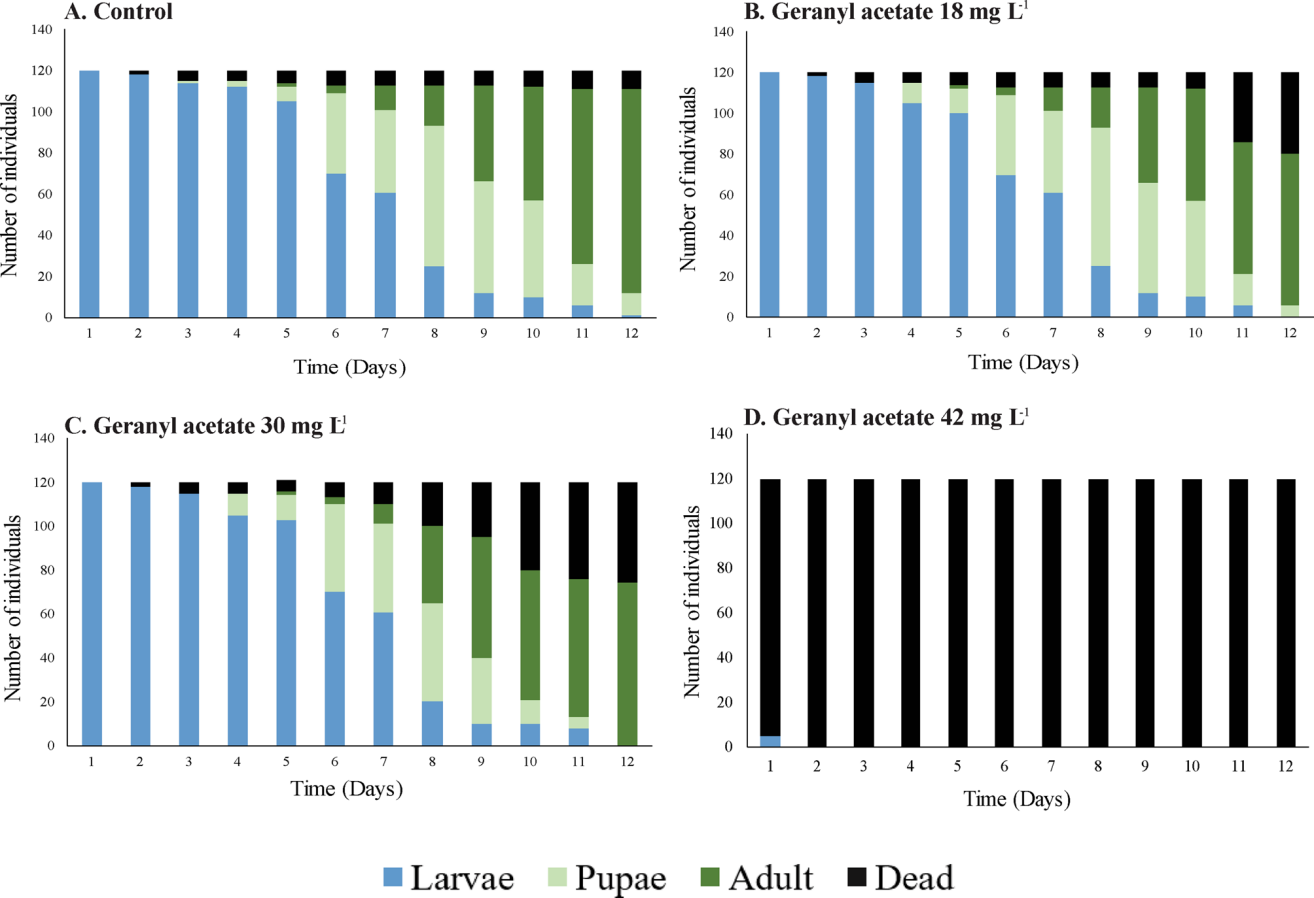
Duration in days of larval development (L3 and L4) as well as pupal and adult stages of *Aedes aegypti*. The individuals were treated with the major compound geranyl acetate.

The larvae treated with the major compound citral (18 and 30 mg L^−1^) exhibited a similar outcome to the control group. Specifically, the life cycle was completed on day 12, and 60% of the larvae reached the pupal stage on day 7 (Fig. 8A). However, for the larvae treated with 42 mg L^−1^ of citral, there was 100% mortality on day 5 of exposure, with none of the larvae reaching the pupal stage (Fig. 8D). The larvae treated with the major compound geranyl acetate (18 and 30 mg L^−1^) also exhibited a similar outcome to the control group. Namely, the individuals reached the adult stage on day 12, and 65% of the larvae reached the pupal stage (Fig. 9A). For the larvae treated with the highest concentration of geranyl acetate (42 mg L^−1^), there was 100% mortality on the first day of treatment (Fig. 9D).

### JH III and MH levels in larvae treated with *C. flexuosus* EO

We measured JH III and MH in L4 larvae by using HPLC coupled with mass spectrometry using methanol as a mobile phase. MH had a 6.77-min retention time (Supplementary Fig. 1A) that corresponds to 481.1 g mol^−1^. JH III had a 9.86-min retention time (Supplementary Fig. 2A) that corresponds to 266.6 g mol^−1^.

We also prepared chromatograms of *Ae*. *aegypti* L4 larvae treated with either methoprene or diflubenzuron (the positive controls) and compared the peaks found for MH and JH III. With methoprene treatment, we observed a greater area under the curve for MH (82% ± 3.4% at 3 mg L^−1^ and 65% ± 0% at 6 mg L^−1^) compared with JH III (18% ± 3.4% at 3 mg L^−1^ and 35% ± 0% at 6 mg L^−1^). Treatment with diflubenzuron produced similar results: a greater area under the curve for MH (80% ± 0% at 3 mg L^−1^ and 81% ± 0% at 6 mg L^−1^) compared with JH III (20% ± 0% at 3 mg L^−1^ and 19% ± 0% at 6 mg L^−1^) (Supplementary Fig. 3).

To analyse the chromatograms of *Ae*. *aegypti* L4 larvae treated with *C. flexuosus* EO and its major compounds (citral and geranyl acetate), we compared the area under the curve for MH and JH III in the positive controls (methoprene and diflubenzuron) with the values observed for treatment with *C. flexuosus* EO (MH: 30% ± 1.1% at 3 mg L^−1^ and 43% ± 0% at 6 mg L^−1^; JH III: 4% ± 2.5% at 3 mg L^−1^ and 22% ± 0% at 6 mg L^−1^), citral (MH: 26% ± 0% at 42 mg L^−1^; JH III: 1% ± 0% at 42 mg L^−1^), and geranyl acetate (MH: 36% ± 0% at 30 mg L^−1^ and 29% ± 0% at 18 mg L^−1^; JH III: 3% ± 0% at 30 mg L^−1^ and 6% ± 0% at 18 mg L^−1^). Overall, treatment with *C. flexuosus* EO and its major compounds (citral and geranyl acetate) markedly reduced the concentration of MH and JH III in *Ae. Aegypti* larvae, with values that were under the limit of detection for this technique (Supplementary Fig. 4).

## Discussion

The mechanism of action of most synthetic insecticides is principally related to four targets: acetylcholinesterase, the voltage-gated chloride channel, the acetylcholine receptor, and the ɣ-aminobutyric acid receptor. The union or interaction of these synthetic molecules triggers a series of biochemical events that modify the physiological functions or cause death^29,30^. However, the action of these molecules is highly specific, contributing to the generation of resistance through the overexpression of detoxifying enzymes and alterations in the target site^31^. Due to the multiple resistance problems generated by the continuous use of synthetic molecules, the search for new molecules that counter the resistance levels has prompted the screening of substances of natural origin like EO and their major compounds^29,32^. These natural substances have wide range of molecular targets, including proteins (enzymes, receptors, ions channels, and structural proteins), nucleic acids, and biomembranes, as well as the ability to interfere in different metabolic pathways^33^. Moreover, natural molecules are biodegradable and cause toxic effects via contact, ingestion, or fumigation^34^. This opens a new opportunity for mosquito control by affecting the life cycle while the larvae develop and avoid the rise of resistant insects.

### Ovicidal activity

One of the major problems in *Ae. aegypti* control is that its eggs are highly resistant to desiccation periods; they remain latent until almost the end of embryonic development^35^. Plants of the genus *Cymbopogon* have been recognised for their ovicidal effect against different species of mosquitoes. Warikoo et al*.*^28^ reported 100% hatching inhibition with *Cymbopogon nardus* EO on *Ae. aegypti* eggs at concentrations of 1%, 10%, and 100%. Additionally, Pushpanathan et al*.*^36^ obtained 22.4% mortality in *Culex quinquefasciatus* eggs treated with 100 mg L^−1^ of *Cymbopogon citratus*, and the mortality of eggs increased to 100% with a concentration of 300 mg L^−1^. The results obtained by these authors are consistent with the results from our present study, where sub-lethal concentrations (6, 18, and 30 mg L^−1^) of *C. flexuosus* EO and their major compounds decreased the hatching of *Ae. aegypti* eggs*.*

The ovicidal effect found in this study could be because *Ae. aegypti* eggs have semi-permeable external (exo-chorion) and internal (endo-chorion) layers, through which some molecules can penetrate. On the inner side of the endo-chorion is a serosal cuticle, which is formed during embryogenesis and protects the embryo from external factors like desiccation or the presence of bacteria or insecticides^35^. However, this serosal cuticle can be disrupted by lipophilic substances^37^ like the terpenes citral and geranyl acetate, the major compounds of *C. flexuosus* EO. In this sense, the hatching inhibition observed in this study appears to be due to an interruption of embryonic development caused by the physiological alterations in water and gas exchange, enzymatic modifications, and hormonal changes^37,38^ (Fig. 2).

On the other hand, when we analysed the hatching inhibition using the major compounds of *C. flexuosus* EO, diflubenzuron, and methoprene, we noted that the inhibition caused by diflubenzuron and citral were similar (40% and 59%). We also noted similar hatching inhibition for methoprene and geranyl acetate (68% and 75%). Diflubenzuron is a chitin synthesis inhibitor in the larval and pupal stages, and it produced greater hatching inhibition than *C. flexuosus* EO (Fig. 2). Moreover, the values are higher than these reported by Suman et al*.*^20^, who treated freshly laid and embryonated *Ae. aegypti* eggs with diflubenzuron concentrations of 0.001, 0.01, 0.1, and 1 mg L^−1^, and showed a hatching inhibition of 25.5%. The mechanism of action of this insecticide is related to the inhibition of transmembrane transport of chitin precursors, preventing the formation of microfibrils and embryonic development^39^. While the effect of methoprene on the embryonic development of hatched *Ae. aegypti* eggs has not been studied, this substance is a JH analogue and can interfere with ecdysone gene expression and alter normal embryonic development^40^.

### Activity on larvae and the life cycle

We found that sub-lethal concentrations of *C. flexuosus* EO affected the normal development of the juvenile phases of *Ae. aegypti*. The EO caused developmental alterations and morphological changes in larvae, represented by the failure to progress past this stage as well as an increase in the size of the larva, the width of the head, and the length of the siphon (Figs. 3 and 4). These results agree with the report by Soonwera and Phasomkusolsil^41^. These authors found morphological abnormalities in *Ae. aegypti* larvae, pupae, and adults and high mortality of larvae that did not reach the pupal stage at concentrations of 1%, 5%, and 10% of *C. citratus* EO*.*

We observed similar morphological changes in larvae treated with *C. flexuosus* EO and methoprene, with an increase in the width of the head and the size of the larva (Figs. 3 and 4). Because methoprene is a JH analogue, it acts directly on molting, permitting the increase in larval size but preventing progression to the pupal stage^42^. This inhibitory effect on the juvenile stage change was also observed in larvae treated with *C. flexuosus* EO: they stayed in the larval stage for a maximum of 12 days. On the other hand, the measures of the morphological parameters in larvae treated with diflubenzuron were lower than the measures of larvae treated with *C. flexuosus* EO (Fig. 6). Diflubenzuron inhibits chitin synthesis, causing alterations in the cuticle layers during molting^11,43^. These changes also alter the normal duration of developmental stages. In our case, they caused inhibition of larval development, with the larvae remaining in that stage for the 12 days of observation.

We also evaluated whether the effects induced by *C. flexuosus* EO are due to the activity of the major compounds (citral and geranyl acetate). We found that each compound increased the LL but not the CD and SL, similar to the effect of by *C. flexuosus* EO. Neither citral nor geranyl acetate caused a notable inhibition on the larval stage development at concentrations of 18 and 30 mg L^−1^. However, at the highest concentration (42 mg L^−1^), both compounds caused larval mortality (Figs. 8 and 9).

The major compound in *C. flexuosus* EO is citral. It is a monoterpene with an acyclic aldehyde functional group and has several biological properties; it is responsible for the aroma in species from the genus *Cymbopogon*. The 100% mortality of *Ae. aegypti* larvae treated with citral (Fig. 8) obtained in the present study is similar to that reported by other researchers^27,43^. Concerning size modifications in larvae, the mechanism of action could be related to the study by Chaimovitsh et al*.*^44^. These authors reported that the principal target of citral is microtubules, which directly interact with tubulin and cause abnormalities to the cell membrane in both animal and plant cells. Additionally, Orhan et al*.*^45^ found that citral is a reversible competitive inhibitor of acetylcholinesterase (AChE), affecting nerve impulse transmission. Finally, Matsuura et al*.*^46^ found that citral has an inhibitory activity against tyrosinase, an enzyme responsible for different biological processes, including exoskeleton consolidation in arthropods during molting^47^.

Concerning the bioactivity observed under geranyl acetate treatment (an acyclic monoterpene), the results of the present study are similar to these reported by Michaelakis et al*.*^48^. These researchers evaluated the repellent and larvicidal activity of different compounds and derivatives on *Ae. aegypti*. They found that although the larvicidal activity of geranyl acetate is minor relative to that of citral, geranyl acetate presented higher repellent and larvicidal activity due to its functionality and degree of saturation. Additionally, Cheng et al*.*^49^ reported that geranyl acetate had 100% larvicidal activity against *Aedes albopictus* larvae. Although the exact mechanism of action involved is unknown, many authors have mentioned that the monoterpene structure and their functional modifications play a fundamental role in the activity against mosquitoes, potentialised in acetylated forms^50^.

Considering these results, we can infer that *C. flexuosus* EO in sub-lethal doses causes a similar effect as the JH analogue, probably altering the homeostasis of hormones involved in molting, which leads to abnormal larval development and growth^51,52^. Rattan^33^ mentioned that EO and its constituents affect biochemical processes of insects, especially endocrine balance, with alterations in morphogenesis. However, this effect is not caused exclusively by the major compounds of the oil, namely citral and geranyl acetate. Hence, there are likely synergic effects involving multiple components of the EO, resulting in a greater biological response^53^.

Due to the potential application of EO and their major compounds against *Ae. aegypti*, it is necessary to know the potential effects in field conditions, especially off-target effects. The data on the cytotoxic effects of these substances has come from in vitro cell viability analysis using primary cells and cell lines^54,55^. For example, citral did not affect the viability of human dermal fibroblasts (HDF) (Inhibition Concentration-IC_50_ = 0.1% v/v), normal spleen cells, and human umbilical vein endothelial cells (HUVEC)^56–58^. However, Souza et al*.*^56^ mentioned that citral exhibited cytotoxicity and genotoxic effects at concentrations ≥ 5 mg mL^−1^ on non-metabolising human hepatic cells (leucocytes) and metabolising human hepatic cells (HepG2). Using geranyl acetate, Portilla et al*.*^59^ did not find mutagenic, tumorigenic, or reproductive risks based on in silico analysis using the Osiris and Molinspiration Cheminformatics programs. Moreover, they reported the low cytotoxicity of this compound on the Vero cell line (normal epithelial cells from the kidney of an African green monkey) and the HepaRG cell line (normal human hepatocytes), with an LC_50_ of 200 μM^60^. Finally, by examining HDF and employing *C. flexuosus* EO, Adukwu et al*.*^58^ obtained an IC_50_ of 0.13% (v/v), which was slightly higher than the IC_50_ of citral. In general, EO whose main compounds are terpenes present great possibilities for insect control due to their low toxicity against mammals and off-target organisms.

We highlight that there are no previous publications describing the evaluation of *C. flexuosus* EO and their major compounds in field conditions and its biological effect in other invertebrates. To achieve field evaluations, it is necessary to improve some physicochemical characteristics of EOs, like high volatility and low availability of the active polyphenolic compounds^61,62^. For this reason, the current research of insecticidal EO is focused on the formulation of these compounds as microemulsions or nanoemulsions^62^. However, before a natural product be commercialized, it must meet requirements regarding the process of obtaining the raw material, the quality control of the final product, and the final application method of the product (considering climatic conditions)^60–64^.

### Measuring MH and JH III levels

With the HPLC technique coupled with mass spectrometry, we identified the signals corresponding to MH and JH III in untreated individuals. With diflubenzuron and methoprene treatments, we did not detect a peak corresponding to JH III. Considering that the treatments were performed in L3 larvae, the absence of JH III may be due to the natural diminution of the concentration of this hormone, which is higher during the initial larval stages and decreases in the L4 stage to permit pupation and metamorphosis^65,66^. When the levels of JH III decrease at the end of the larval stage, MH induces the change from larva to pupa and from pupa to adult^67^. Corroborating this fact, Hernández-Martínez et al*.*^65^ measured JH III changes during the gonotrophic cycle of *Ae. aegypti*, without detecting the release of methyl farnesoate, an immediate precursor of JH, in *Ae. aegypti* adults, but they did detect it in the larval stage.

When we examined the chromatogram corresponding to L4 larvae treated with *C. flexuosus* EO and its major compounds (citral and geranyl acetate), there was no clear peak corresponding to MH or JH III or any of their fractions. Considering the diminution of JH III in L4 larvae and the possible effect of the EO on larval development, the concentration of this hormone could have been below the limit of detection based on the calibration curves performed for JH and MH. Regarding MH, the process of ecdysis and chitin formation could be altered due to the activity of citral component (the major component of the *C. flexuosus* EO), causing changes in the concentration of this hormone.

## Conclusions

The EO from *C. flexuosus* used at sub-lethal concentrations had bioactivity in eggs and larvae of *Ae. aegypti*. In the egg stage, geranyl acetate (a major compound of EO) altered the development of the embryo, decreasing the number of hatched individuals, likely by penetrating the chorion. In the early larval stages (L1, L2, and L3), the EO caused an effect similar to a JH analogue, lengthening the larval period and making pupation impossible. Additionally, the major component citral could alter ecdysis, a process directly related to MH. Using the HPLC technique coupled with mass spectrometry, we identified the signal corresponding to JH III and MH in untreated individuals. However, in larvae treated with *C. flexuosus* EO and its major compounds (citral and geranyl acetate), there was not a clear peak for either hormone. This result demonstrates an alteration in the concentration of JH III and MH due to the activity of the EO, an effect that cannot be attributed exclusively to the major components of EO.

## Materials and methods

### Essential oil extraction and isolation of major compounds

*C. flexuosus* was collected from the department of Santander (Colombia) at Complejo Agroindustrial Piloto (Centro Nacional de Investigación para la Agroindustrialización de Plantas Aromáticas y Medicinales Tropicales [CENIVAM]), under permission to conduct this research from the Ministerio de Ambiente y Desarrollo Sostenible (MADS), through its Dirección de Bosques, Biodiversidad y Servicios Ecosistémicos and access to genetic resources and derived products for the program the Unión Temporal Bio-Red-CO-CENIVAM (Resolution 0812, June 4, 2014). Contract No. 270 for the access to genetic resources and derived products for bioprospecting purposes, signed between the Environment and Sustainable Development Ministry and the Industrial University of Santander. Authorization for the collection of wild species specimens from biological diversity for non-commercial scientific research purposes granted by the National Environmental Licensing Authority—ANLA (Resolutions 004 22 of January 2015 and 0260 March 11, 2016). The material was identified up to the species level, and the exsiccatae were deposited in the National Herbarium with Voucher No. 519986. This approach complies with local and national regulations.

*Cymbopogon flexuosus* EO was provided and characterised by the Centro de Investigación en biomoléculas (CIBIMOL) and CENIVAM, of the Universidad Industrial de Santander (Colombia). The oil extraction methodology, as well as its chemical characterisation, was carried out following the methodology described by Stashenko et al*.*^68^. The essential oil was obtained by hydrodistillation (HD) and microwave-assisted hydrodistillation (MWHD). The components were identified by comparing their relative retention times and mass spectrometry with standard compounds^69^. The EO chemical characterisation has been described by Vera et al.^21^: citral (geranial 37.5% and neral 28.2%), geranyl acetate (10.0%), geraniol (9.0%), and β-bourboneno trans-β-cariofilene (2.0%). The major compounds (citral and geranyl acetate) were commercially acquired from Sigma-Aldrich (St. Louis, MO, USA).

### Biological material

The experiments were performed with a colony of *Ae. aegypti*, Rockefeller strain, maintained in an insectary at 25 ± 5 °C, with relative humidity of 70% ± 5%, and a 12-h photoperiod. Male adults were fed with a sugar solution of 10% honey according to the breeding protocol for this species. Mosquito females were blood-fed for 15 min with an albino Wistar rat (Rattus norvegicus) (WI IOPS AF/Han strain). During the procedure law 84 of 1989 of chapter VI, articles 23 (literal a-b-c) and 24 of the National Statute for the Protection of Animals and that regulates the use of live animals in research experiments complied. Wistar rats were used only for this procedure, provided by the bioterium of the Universidad Industrial de Santander, complying with the laboratory animal handling procedures and under the approval of the Ethics Committee (CEINCI; Minutes No. 22, Dec 6, 2019). Furthermore, the study was carried out in compliance with the ARRIVE guidelines.

Once the females were fed, glass cups with Whatman No 1 filter papers folded as a cone were placed inside the breeding cages to internally coat the walls of the vessel and allow the collection of eggs. To synchronise egg hatching, the filter paper was removed after the embryo maturation process (72 h) and then allowed to dry for 3 days at room temperature. Subsequently, hatching was stimulated by immersing the eggs in dechlorinated water. The larvae were kept in plastic trays and fed with TetraMin Tropical Flakes fish concentrate.

### Ovicidal activity

We used the methodology of Suman et al*.*^20^ to assess the ovicidal effect of the tested compounds. The sub-lethal concentration was established based on LC calculated for *C. flexuosus* EO in the fourth early-stage larvae of *Ae. aegypti* by Vera et al*.*^21^ at 24 h (LC_50_ = 17.2 mg L^−1^; LC_95_ = 49.9 mg L^−1^) and 48 h (LC_50_ = 14.7 mg L^−1^; LC_95_ = 55.6 mg L^−1^), settling on 6, 18, and 30 mg L^−1^. We evaluated the major compounds (citral and geranyl acetate) of *C. flexuosus* EO. Also, we evaluated methoprene (PESTANAL; Sigma-Aldrich) and diflubenzuron (PESTANAL; Sigma-Aldrich) as positive control and dimethyl sulphoxide (DMSO 0.5%) as a negative control.

We separated gravid *Ae. aegypti* females in a security cage. Inside the cage, we placed a cup that was coated inside with half of a Whatman No 1 filter paper. When the females oviposited, the eggs were counted and examined under a stereoscope to verify integrity (re-collected after 72 h). For each evaluated substance and concentration, we randomly selected 80 embryonated eggs and transferred them to individual plastic containers with 49.5 mL of dechlorinated water and 500 µL of substance at the established concentrations (6, 18, and 30 mg L^−1^). The hatching percentage of the eggs was evaluated up to 120 h after submerging the obtained eggs in dechlorinated water. The emerged first instar larvae were counted under a microscope, and eggs that did not hatch after 7 days were considered not viable. The hatching value was estimated as the percentage of eggs that went on to the larval stage.

### Larvicidal activity

For larvicidal activity experiments, we established two sub-lethal concentrations of 3 and 6 mg L^−1^ of *C. flexuosus* EO. To evaluate the major compounds citral and geranyl acetate, we also investigated the concentrations of 3 and 6 mg L^−1^. However, we did not observe any effect on the larvae. For this reason, we increased the concentrations until establishing three sub-lethal concentrations of 18, 30, and 42 mg L^−1^. Methoprene and diflubenzuron at 3 and 6 mg L^−1^ were used as positive controls and DMSO (0.5%) as a negative control.

To determine the effect on larval development, we used the methodology by Leyva et al.^70^ with some modifications. Briefly, 10 L3 larvae were selected and transferred by Pasteur pipettes to 200-mL plastic cups with 99.5 mL of dechlorinated water and 0.5 mL of each treatment (negative control, *C. flexuosus* EO, citral, geranyl acetate, methoprene, or diflubenzuron). All larvae were supplied with 2% fish feed in chlorine-free water to ensure their survival. After 24 h of treatment, the dead larvae were removed, and the survivors remained in the water until they pupated. At 48 h, five larvae were taken for each treatment and were used to measure morphological parameters (in mm): LL, CD, and SL. Nine replicates were used for each concentration by substance (EO, citral, geranyl acetate, diflubenzuron and methoprene), distributed on three different days.

### Effects on MH and JH III

To evaluate changes in MH and JH III, 200 newly emerged L3 larvae were selected and treated with *C. flexuosus* EO at 3 and 6 mg L^−1^, and with citral and geranyl acetate at 18, 30, and 42 mg L^−1^. For this experiment, molecular grade standards of methoprene and diflubenzuron were used at 3 and 6 mg L^−1^ (Fig. 1).

After 24 h, the larvae of each treatment were extracted and homogenised in 2 mL of a 50 mM phosphate-buffered saline (pH 7.4) using a BeadBug Mini Homogeniser (Benchmark, model D1030). The homogenate obtained was filtered on glass wool to remove impurities and subsequently analysed by HPLC coupled with mass spectrometry. Nine replicates of this experiment were performed for each concentration of each substance, with their respective calibration curve, using MH and JH (Sigma-Aldrich) as standards. We adapted the methodology of Zhou et al*.*^69^ for the quantification of JH and HM by HPLC coupled with mass spectrometry (electrospray ionisation–ion trap analyser). The following set-up was used: an Elite LabChrom liquid chromatography apparatus (Hitachi, VWR) with a detection range of 190–600 nm (ultraviolet [UV] light), an injection volume of 0.1–90 μL, and a flow range of 0.001–10 mL/min, coupled to a mass spectrometer, with a Zorbax XDB-C18 octadecylsiloxane column (4.6 mm × 10 mm, 2.0 μm internal diameter).

Water (0.1% formic acid) plus acetonitrile (0.1% formic acid) and water (0.1% formic acid) plus methanol (0.1% formic acid) were used as the mobile phase, applying a binary gradient at a flow of 0.30 mL min^−1^, with an injection volume of 10 μL. The total running time was 15 min. The retention time corresponding to the signal of each hormone was obtained, and the concentration levels of the hormones in each treatment were determined.

### Statistical analyses

All data were subjected to normality tests. The fixed factors in each experiment were the substances (*C. flexuosus* EO, citral, geranyl acetate, diflubenzuron, and methoprene) and the concentrations evaluated. The random factors in each experiment were the different effects of the substances on *Ae. aegypti*: percentage of hatched eggs, morphological parameters of larvae, and percentage of individuals in each developmental stage. When the data presented a normal distribution, we used an analysis of variance (ANOVA) and, subsequently, Tukey’s test. If the distribution was not normal, we applied the non-parametric Kruskal–Wallis test. A *p* value ≤ 0.05 was considered statistically significant. The results were analysed with Statistica version 11 (TIBCO Software, Inc., Palo Alto, CA, USA).

## Supplementary Information

Supplementary Information.
