# Supplementary material for: Impact of Cymbopogon flexuosus (Poaceae) essential oil and primary components on the eclosion and larval development of Aedes aegypti
Source: Sci Rep. 2021 Dec 21;11:24291. doi: 10.1038/s41598-021-03819-2 (PMC8692593; doi:10.1038/s41598-021-03819-2)
Supplement: Supplementary file 1 — Supplementary Information. [file 41598_2021_3819_MOESM1_ESM.docx]

**Impact of *Cymbopogon flexuosus* (Poaceae) essential oil and primary components on the eclosion and larval development of *Aedes aegypti***

Ruth Mariela Castillo-Morales^1^, Sugey Ortiz Serrano^1^, Adriana Lisseth Rodríguez Villamizar^2^, Stelia Carolina Mendez-Sanchez^2^*, Jonny E. Duque^1^*

1. Centro de Investigaciones en Enfermedades Tropicales - CINTROP. Facultad de Salud. Escuela de Medicina, Departamento de Ciencias Básicas, Universidad Industrial de Santander, Guatiguará Technology and Research Park, Km 2 Vía El Refugio, Piedecuesta, Santander, Colombia. Phone number: 57-7-6344000 Ext. 3503

2. Grupo de Investigación en Bioquímica y Microbiología (GIBIM). Escuela de Química, Universidad Industrial de Santander, Bucaramanga A.A. 678, Colombia

*Corresponding Author: [jonedulu@uis.edu.co](mailto:jonedulu@uis.edu.co)


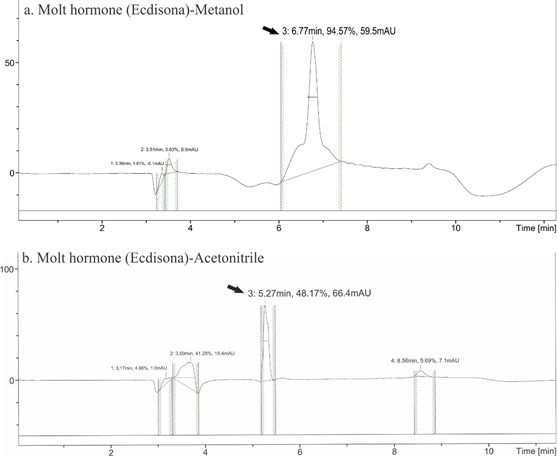


**Supplementary Figure 1.** Molt Hormone (MH) HPLC-Mass spectrometry chromatograms *Aedes aegypti* larvae (L4) without treatment. Y-axes: signal intensity (mAU). X-axes: retention time in minutes. **a.** Methanol-water mobile phase chromatogram. The retention time of 6.77 min, molecular mass detected by the mass spectrometer: 481.1 g.mol^-1^.


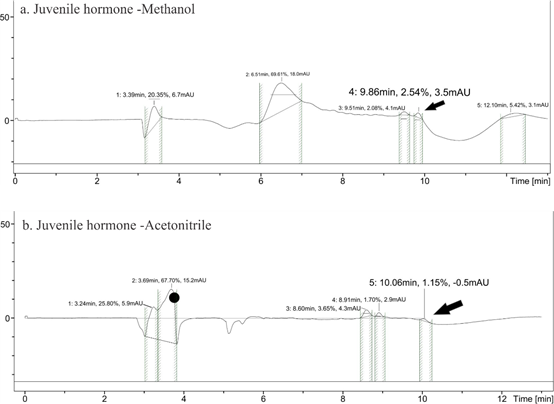


**Supplementary Figure 2.** Juvenile hormone III (JH III) HPLC-Mass spectrometry chromatograms *Aedes aegypti* larvae (L4) without treatment. Y-axes: signal intensity (mAU). X-axes: retention time in minutes. a. Methanol-water mobile phase chromatogram. The retention time of 9.86 min. b. Acetonitrile (ACN)-water mobile phase chromatogram. The retention time of 10.06 min, molecular mass detected by the mass spectrometer: 481.1 g.mol^-1^.

**
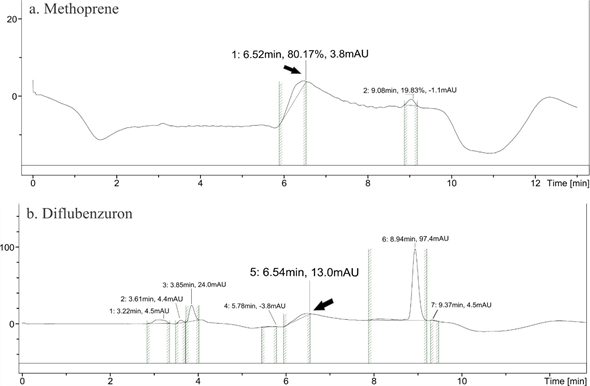
**

**Supplementary Figure 3.** Methoprene and Diflubenzuron HPLC-Mass spectrometry chromatograms *Aedes aegypti* larvae (L4). Y-axes: signal intensity (mAU). X-axes: retention time in minutes. a. *Aedes aegypti* larvae (L4) chromatogram treated with Methoprene. The retention time of 6.52 min and molecular mass detected by the mass spectrometer of 481.0 g.mol^-1^, corresponding to MH. b. *Aedes aegypti* larvae (L4) chromatogram treated with Diflubenzuron. The retention time of 6.54 min, molecular mass detected by the mass spectrometer of 481.1 g.mol^-1^, corresponding to MH.

**
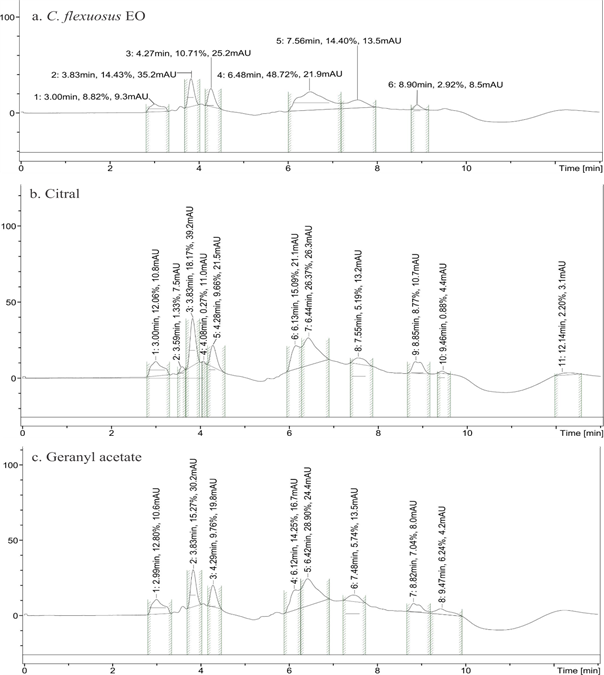
**

**Supplementary Figure 4.** Major compounds (Citral and Geranyl acetate) HPLC-Mass spectrometry chromatograms *Aedes aegypti* larvae (L4) treated with *C. flexuosus* EO and its major compounds (Citral and Geranyl acetate). Y-axes: signal intensity (mAU). X-axes: retention time in minutes. Don´t detect any titter corresponding to MH or JH III. **a.** *Aedes aegypti* larvae (L4) chromatogram treated with *C. flexuosus* EO. **b.** *Aedes aegypti* larvae (L4) chromatogram treated with the major compound Citral. **c.** *Aedes aegypti* larvae (L4) chromatogram treated with the major compound Geranyl acetate.
